# PTK7-Targeting CAR T-Cells for the Treatment of Lung Cancer and Other Malignancies

**DOI:** 10.3389/fimmu.2021.665970

**Published:** 2021-08-12

**Authors:** Yamin Jie, Guijun Liu, Lina Feng, Ying Li, Mingyan E, Liangliang Wu, Yinyin Li, Guanghua Rong, Yongwu Li, Huafeng Wei, Anxin Gu

**Affiliations:** ^1^Department of Radiation Oncology, The Fourth Affiliated Hospital of Harbin Medical University, Harbin, China; ^2^The Second Affiliated Hospital of Heilongjiang University of Chinese Medicine, Harbin, China; ^3^Department of Radiation Oncology, Harbin Medical University Cancer Hospital, Harbin, China; ^4^Institute of Hard Tissue Development and Regeneration, The Second Affiliated Hospital of Harbin Medical University, Harbin, China; ^5^Key Lab of Cancer Center, General Hospital of Chinese PLA & Beijing Key Laboratory of Cell Engineering & Antibody, Beijing, China; ^6^Liver Cancer Unit, Department of Liver Disease, The Fifth Medical Center of PLA General Hospital, Beijing, China; ^7^Department of Radiology, The Fifth Medical Center of PLA General Hospital, Beijing, China

**Keywords:** chimeric antigen receptor (CAR), cancer stem cells (CSCs), lung cancer 4, adoptive cell therapy (ACT), tumor-initiating cells (TICs), PTK7

## Abstract

In spite of impressive success in treating hematologic malignancies, adoptive therapy with chimeric antigen receptor modified T cells (CAR T) has not yet been effective in solid tumors, where identification of suitable tumor-specific antigens remains a major obstacle for CAR T-cell therapy due to the “on target off tumor” toxicity. Protein tyrosine kinase 7 (PTK7) is a member of the Wnt-related pseudokinases and identified as a highly expressed antigen enriched in cancer stem cells (CSCs) from multiple solid tumors, including but not limited to triple-negative breast cancer, non-small-cell lung cancer, and ovarian cancer, suggesting it may serve as a promising tumor-specific target for CAR T-cell therapy. In this study, we constructed three different PTK7-specific CAR (PTK7-CAR1/2/3), each comprising a humanized PTK7-specific single-chain variable fragment (scFv), hinge and transmembrane (TM) regions of the human CD8α molecule, 4-1BB intracellular co-stimulatory domain (BB-ICD), and CD3ζ intracellular domain (CD3ζ-ICD) sequence, and then prepared the CAR T cells by lentivirus-mediated transduction of human activated T cells accordingly, and we sequentially evaluated their antigen-specific recognition and killing activity *in vitro* and *in vivo*. T cells transduced with all three PTK7-CAR candidates exhibited antigen-specific cytokine production and potent cytotoxicity against naturally expressing PTK7-positive tumor cells of multiple cancer types without mediating cytotoxicity of a panel of normal primary human cells; meanwhile, *in vitro* recursive cytotoxicity assays demonstrated that only PTK7-CAR2 modified T cells retained effective through multiple rounds of tumor challenge. Using *in vivo* xenograft models of lung cancers with different expression levels of PTK7, systemic delivery of PTK7-CAR2 modified T cells significantly prevented tumor growth and prolonged overall survival of mice. Altogether, our results support PTK7 as a therapeutic target suitable for CAR T-cell therapy that could be applied for lung cancers and many other solid cancers with PTK7 overexpression.

## Introduction

Chimeric antigen receptor (CAR)-modified T-cell (CAR T-cell) therapy is an innovative immunotherapeutic approach that vigorously rejuvenates the long-term pursuit adoptive cell transfer (ACT) for cancer immunotherapy (1, 2). Typical synthetic CAR comprises of single-chain variable fragment (scFv) of a monoclonal antibody (mAb), hinge/spacer and transmembrane (TM), and co-stimulatory and activating signaling domains from one or two co-stimulatory molecules and CD3ζ chain of the T cell receptor (TCR) complex respectively (1, 3). CAR modification confers T cells with “*de novo*” defined antigen specificities independently of both the natural TCR and major histocompatibility complex (MHC) restriction, which not only overcomes the downregulation of Human Leucocyte Antigen (HLA, human MHC) molecules frequently observed in cancer cells, but also widens the repertoire of actionable targets due to scFv-mediated antigen recognition of non-protein epitopes, thus greatly expanding the potentials of ACT for cancer immunotherapy (1, 3, 4). CAR T-cell therapy targeting CD19 antigen has achieved a remarkable therapeutic efficacy in treating relapse or refractory B-cell malignancies, culminating in the regulatory approval of two CAR T-cell products for patients with certain leukemia and lymphoma (1, 2); in addition, CAR T cells targeting other antigens, such as BCMA and CD22, have also exhibited a promising therapeutic potential in treating some type of intractable leukemia and multiple myeloma (5–7). These results have demonstrated that CAR T cells can be artificially generated with desirable characteristics to induce durable and complete responses in cancer patients even with highly refractory disease.

Despite great success in treating hematological malignancy, CAR T-cell therapy in solid tumor is still in its infancy with scant objective response seen (4). Among various factors constraining the efficacy of CAR T-cell therapy in solid tumor, a major obstacle is the lack of appropriate tumor antigens suitable for CAR-T targeting (4, 8). At present, the majority of CAR T-cell targets in solid tumors are overexpressed tumor-associated antigens (TAA) with lower-level expression in normal tissues as compared to tumor tissues, such as HER2, GPC-3, EGFR, mesothelin, PSMA, and IL13Ra2, which greatly limits the maximum safety dosage in order to avoid on-target off-tumor side effect and consequently results in unsatisfactory clinical efficacy (1, 8, 9). In addition, due to the extreme heterogeneous antigen expression and highly genomic instability in solid tumors, tumor cells are prone to produce antigen-loss variants under the immune selection pressure from CAR T-cell therapy, leading to immune escape (8). Therefore, the identification of new target antigens that are not easy to generate immune escape is still a key issue for the successful treatment of solid tumors with CAR T cells.

PTK7, also known as colon carcinoma kinase 4 (CCK-4), is a member of the pseudokinase family of receptor tyrosine kinases (RTKs) that have an intracellular catalytically inactive tyrosine kinase-like domain (10, 11). PTK7 is expressed during embryogenesis but absent from normal vital adult tissues, apart from a subset of immature CD4^+^ recent thymic emigrants (RTEs) and plasmacytoid dendritic cells (pDCs) and low-level expression on some normal tissues (10–12). Genetic and biochemical studies have demonstrated an involvement of PTK7 in non-canonical Wnt signaling *via* interacting with Wnt ligands such asROR2, Wnt5a, or Wnt3a (13, 14). PTK7 is strongly associated with planar cell polarity (PCP) regulation as PTK7-deficient embryos exhibit severe developmental defects in PCP (15, 16). In addition, evidence is also present for context-dependent roles of PTK7 in the vascular endothelial growth factor (VEGF), semaphorin/plexin, and canonical Wnt signaling pathways (11). Oncogenic functions of PTK7 have been documented in several hematological and solid tumors (10, 11). Recent studies showed that PTK7 is overexpressed in triple-negative breast cancer (TNBC), non-small-cell lung cancer (NSCLC), ovarian cancer (OVCA), cervical cancer, esophageal squamous cell carcinoma (ESCC), and hepatocellular carcinoma (HCC) and enriched in tumor-initiating cells (TICs) from TNBC, OVCA, and NSCLC patient-derived xenografts (PDXs), and its overexpression is associated with poor survival in NSCLC, cervical cancer, ESCC, and HCC (12, 17–22). Bie J et al. found that PTK7 was dramatically upregulated in the ESCC tissues and cancer stem cell (CSC)-like cells and its knockdown reduced sphere formation, promoted apoptosis, and suppressed invasive behavior of tumor cells (17). Chen et al. conducted a large-scale meta-analysis to search the genes specifically overexpressed in lung adenocarcinoma where PTK7 was identified to be the one of overexpressed six genes confirmed by IHC analysis in primary adenocarcinoma samples. Functional investigation revealed that PTK7 knockdown decreased cell viability and increased apoptosis in lung adenocarcinoma cell lines. More importantly, a PTK7-targeting antibody-drug conjugate (ADC) induced sustained tumor regressions in lung and breast tumor xenograft models (12); furthermore, recent studies have documented the success and feasibility of PTK7-based tumor-targeting strategies by using PTK7-specific antibodies or aptamers for *in vivo* imaging or drug delivery (23–25). These pioneering studies strongly support the potential of the PTK7 as an attractive candidate for CAR T-cell therapy that could be broadly applied.

In this study, we developed an alternative approach of exploiting PTK7 as a target for CAR T-cell therapy. The rationale is based in part upon the hypothesis that PTK7 expression is enriched on TIC/CSC-like cells, and targeting antigens with enriched expression in TIC/CSC-like cells would achieve a long-term antitumor effect (26). Given the predicted potential and safety of PTK7 as an immunotherapy target, we sought to develop PTK7-specific CAR T-cell therapy for lung cancer and to evaluate its efficacy and safety in *in vitro* and *in vivo* preclinical models.

## Materials and Methods

### Cell Lines

Human NSLCL cell lines H520, H1975, and H1299, SCLC cell lines H446 and H69, pancreatic cancer cell line BxPC3, breast cancer cell line MDA-DB-468, ovarian cancer cell line OVCAR3, CHO, and HEK-293 T cells were purchased from American Type Culture Collection (ATCC) and maintained in DMEM medium (Thermo Fisher Scientific) supplemented with 10% heat-inactivated fetal bovine serum (FBS), 2 mM glutamine, and 1% penicillin/streptomycin (all from Thermo Fisher Scientific); and all cell lines were cultured at 37°C in a humidified chamber with 5% CO2. Stably transfected PTK7-CHO cell line was constructed by infecting parental CHO cells with lentiviral supernatants containing PTK7 gene (#HG19399-UT, Sino Biological) and sorting for PTK7 expression by using MoFloTM XDP cell sorting system (Beckman Coulter). These cell lines were also infected with the lentiviral supernatants containing Luciferase-IRES-GFP (GL) and were then sorted for GFP expression to obtain GL-expressing cell lines. Human primary normal epithelial cell lines (Mammary, Small Airway, and Renal Epithelial Cells) and human umbilical vein endothelial cells (HUVECs) were obtained from PriCells (Wuhan, China) and cultured according to the supplier’s instructions.

### PTK7-CAR Construction

Sequences of three humanized mouse antihuman PTK7 antibodies (Hu23, Hu24, and Hu58 with the affinity of 3.9, 1.2, and 2.1 nM, respectively) were obtained from a US patent (US20150315293A1). The variable region sequences of heavy (VH) and light chain (VL) of these antibodies were used to design scFv with the sequence of VH-(G_4_S)3 Linker-VL. PTK7-CARs containing scFv from Hu23, Hu24, and Hu58 were designated as PTK7-CAR1, PTK7-CAR2, and PTK7-CAR3, respectively. From the 5′-end to 3′-end, each CAR is comprised of the human CD8α signal peptide sequence, PTK7-scFv, hinge and TM regions of the human CD8α molecule, 4-1BB intracellular domain sequence (BB-ICD), and CD3ζ intracellular domain sequence (CD3ζ-ICD) as previously described (27). Following CAR, a truncated tEGFR sequence is included *via* T2A ribosomal skipping sequence in the construct to allow for potential enrichment, tracking, and depletion if needed of transduced T cells (28). DNA encoding the CARs was codon-optimized and synthesized by General Biosystems (Anhui, China) with appropriate restriction sites. The CAR sequences were then cloned into third-generation self-inactivated lentiviral vector pLVEF derived from pRRLSIN.cPPT.PGK-GFP.WPRE vector (Plasmid #12252, Addgene) with replacing its original human PGK promoter with human EF1α promoter from pWPXLd vector (Plasmid #12258, Addgene). As a negative control, lentiviral vector encoding truncated tEGFR was constructed.

### Lentivirus Production

High-titer replication-incompetent lentiviruses were produced and concentrated as described previously (29). Briefly, HEK-293 T cells were transfected with pVSV-G (VSV glycoprotein expression plasmid), pRSV-Rev (Rev expression plasmid), pMDLg/p.RRE (Gag/Pol expression plasmid), and pLVEF transfer plasmid using polyethylenimine (PEI, Sigma). The viral supernatant was harvested at 24 and 48 h after transfection and concentrated by using Lenti-X Concentrator (Clontech) in accordance with the manufacturer’s instructions.

### CAR T-Cell Production

Human PBMCs were obtained from healthy donors under protocols approved by the Institutional Review Board of Harbin Medical University and isolated by density gradient centrifugation over Ficoll-Paque (GE Healthcare). Freshly isolated PBMCs were then activated with antihuman CD3/CD28 Dynabeads (Thermo Fisher Scientific) at a 3:1 ratio for 48 h followed by two sequential transductions with lentiviruses on RetroNectin-coated non-tissue treated plates and maintained in culture in RPMI-1640 (Thermo Fisher Scientific) supplemented with 10% FBS (Thermo Fisher Scientific) and recombinant human IL-2 (300 U/ml). Fresh media containing cytokine were replenished every other day to maintain T-cell concentration at 0.5×10^6^ cells/ml. Five days after transduction, the CD3/CD28 Dynabeads were removed from the culture by magnetic separation, and CAR T cells were propagated for 14 days in total before using for functional assays. To track T cell numbers over time, viable cells were counted using trypan blue.

### Flow Cytometry

PTK7 expression on tumor cells was detected by mouse monoclonal anti-PTK7 antibody (clone OTI2E7, Invitrogen) and goat anti-mouse IgG-phycoerythrin (PE)-conjugated antibody (Jackson ImmunoResearch). CAR expression on 293T cells was detected by APC-conjugated rabbit monoclonal anti-EGFR antibody (Clone E01, Sino Biological) and biotin-conjugated goat antihuman IgGF(ab′)2 (Jackson ImmunoResearch) and streptavidin-PE (BioLegend). CAR expression on T cells was detected by BV510-conjugated CD3 (clone UCHT1), APC-Cy7-conjugated CD4 (clone OKT4), and FITC-conjugated rabbit monoclonal anti-EGFR antibody (Clone E01, Sino Biological) and biotin-conjugated goat antihuman IgGF(ab′)2 and streptavidin-APC (BioLegend). The phenotype and effector molecule expression on CAR T cells were detected with a panel of monoclonal antihuman antibodies as follows: BV510-conjugated CD3 (clone UCHT1), BV421-conjugated CD4 (clone OKT4), APC-Cy7-conjugated CD8 (clone SK1), FITC-conjugated rabbit monoclonal anti-EGFR antibody, APC-conjugated CD45RO (clone UCHL1), PE-conjugated CCR7 (clone G043H7), PE-conjugated TIM-3 (clone F38-2E2), APC-conjugated PD-1 (clone EH12.2H7), and PE-conjugated Granzyme B (clone GB11, all from BioLegend). CAR T cells in peripheral blood from tumor-bearing tumor were detected by BV510-conjugated CD3 and APC-conjugated rabbit anti-EGFR antibody. In most assays, cells were stained with Zombie Aqua™ Fixable Viability Kit (BioLegend) to exclude dead cells from analysis. Flow cytometry data were acquired with a FACSCantoTM system (BD Biosciences) using DIVA software according to the manufacturers’ instructions.

### Cytokine Release Assays

Control or PTK7-CAR T cells (1×10^5^ cells/100 μl media) were co-cultured with an equal number of target cells for 24 h, after which cell-free supernatants were harvested for testing IL-2 and IFN-γ secretion by ELISA kits (R&D Systems) according to the manufacturer’s instructions.

### Proliferation Assay

Control or CAR T cells were first labeled with 5 μM fluorescent dye carboxyfluorescein diacetate succinimidyl ester (CFSE; Invitrogen) according to the manufacturer’s instructions, and then co-cultured with tumor cells at an effector-to-target ratio of 1:1. CFSE dilution was measured on gated T cells on day 3 using flow cytometry.

### *In Vitro* Killing Assays

For tumor cell killing assays, GL-expressing target cells (1×10^4^ cells/100 μl media) were co-cultured with control or PTK7-CAR T cells at the varying effector-to-target ratios in triplicate wells of white 96-well plates. In some assays, it was conducted in the presence of soluble PTK7 protein (OriGene). Target cell viability was monitored 18 h later by using Bright-Glo™ Luciferase Assay System (Promega) according to the manufacturer’s instructions. The percent lysis (%) was calculated by using the following equation: 1-[bioluminescence value in sample well (target cells + CAR T cells)/maximum bioluminescence value (target cells alone)].

For human primary normal cell killing assays, target cells were first labeled with 5 μM fluorescent dye CFSE according to the manufacturer’s instructions, and then co-cultured with control or PTK7-CAR T cells at the indicated effector-to-target ratios in triplicates. After 18 h incubation at 37°C, mixed cells were harvested and stained with 7-AAD and then subjected to flow cytometric analysis to quantify remaining live (7-AAD negative) target cells. The cytotoxicity was calculated as 100%—the percentage of alive target cells/alive target cells in control wells without effectors.

### *In Vitro* Recursive Cytotoxicity Assays

GL-expressing tumor cells (1×10^5^ cells/500 μl CAR-T culture media) were seeded in 24-well tissue culture plates. After overnight plating, 2.5 × 10^4^ (effector-to-target ratio of 1:4) CART cells in 500 μl media were added to the monolayer of tumor cells (round 1). Three days later, when most of the target tumor cells were confirmed to be killed by trypan blue staining, all cells in the well were collected and washed with PBS, resuspended in fresh medium, and added to a new plate seeded with tumor cells for 3 days (round 2). This procedure was repeated one more time, if applicable (round 3). At the end of each round, a duplicate well was harvested for counting of residual tumor cells (GFP^+^) and CAR T cells (CD3^+^EGFR^+^) and other phenotypic analysis (granzyme B, PD-1, TIM-3) of CAR T cells by flow cytometry.

### *In Vivo* Tumor Models

All animal experiments were conducted under a protocol approved by the Institutional Animal Care and Use Committee of the Harbin Medical University. Six- to 8-week-old B-NSG mice (NOD-Prkdcscid Il2rgtm1/Bcgen) were obtained from Biocytogen Co., Ltd (Beijing, China) and maintained on a 12 h light-dark cycle in a temperature-controlled high-barrier facility with free access to food and water and treated under specific pathogen-free conditions at the Animal Centre of the Harbin Medical University. The tumor xenograft model was established by subcutaneous (s.c.) inoculation with 3 × 10^5^ H520 or H69 tumor cells suspended in 100 µl PBS. After 7 days, when the tumor was consistently palpable (50–100 mm^3^), mice were randomized into three groups (three to five mice per group) and intravenously (i.v.) injected with 5 × 10^6^control or PTK7-CAR T cells suspended in 100 µl PBS and repeated once 1 week later. Mice were weekly monitored for tumor growth by using a caliper for 60 days, and then euthanized by cervical dislocation with blood and tumor harvested for analysis when they seemed moribund or their tumors reached 15 mm in diameter. Tumor volume (V) was calculated according to the following formula: V (mm^3^) = 0.5 × length × width^2^.

### Immunohistochemistry (IHC)

Tumor tissues were fixed with formalin and embedded in paraffin. Then, 4 mm thick sections were deparaffinized with xylene and rehydrated in decreasing concentrations of ethanol. After heat-induced antigen retrieval, slides were then blocked by 3% BSA and stained with rabbit monoclonal antihuman CD3ϵ antibody (clone SP162, Abcam) or rabbit polyclonal anti-PTK7 antibody (Invitrogen) in the blocking solution overnight at 4°C. Commercially available normal human tissue microarray (TMA; Shanghai Outdo Biotec) including 20 normal tissues (two to four sections per tissue) were retrieved by EDTA solution (Solarbio) and then stained with the same polyclonal anti-PTK7 antibody (1:200 dilution at 4°C overnight). Slides were then rinsed with Tris-HCl/0.05% Tween-20 buffer and visualized with a horseradish peroxidase (HRP)-conjugated anti-rabbit EnVision+ Kit (Dako). PBS substituted for the primary antibody was used as the negative control.

### Statistical Analysis

Statistical analyses were performed with GraphPad Prism software (version 7.0). Differences in groups were determined by two-way ANOVA with Tukey’s multiple comparison test with P<0.05 considered to be a statistically significant. The survival curves were constructed using the Kaplan-Meier method and analyzed by using a log-rank test. All values and error bars represent the mean ± SEM. In the figures, significance of findings was defined as follows: p > 0.05; *p < 0.05; **p < 0.01; ***p < 0.001, or ****p < 0.0001.

## Results

### Generation of PTK7-CAR T Cells

**Figure 1:**
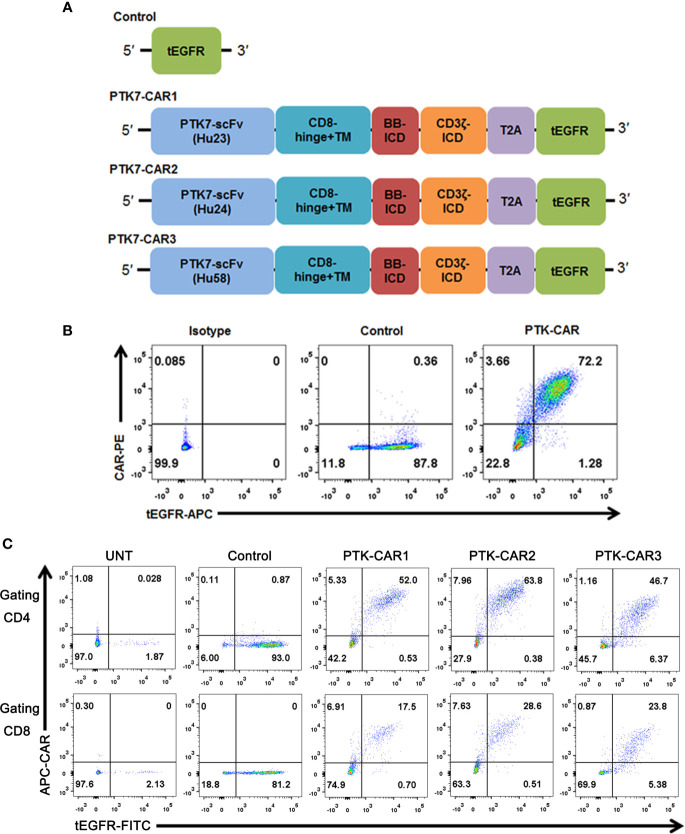
PTK7-CAR generation, cell-surface expression, and transduction of human T cells. **(A)** PTK7-CAR was generated by fusing PTK7-specific scFv to the co-stimulatory signaling domain of the 4-1BB (BB-ICD) and activating signaling domain of CD3ζ (CD3ζ-ICD), a T2A ribosomal skipping sequence, and tEGFR was included for the detection of CAR-modified T cells. **(B)** 293T cells transfected with control or PTK7-CAR constructs express both CAR and the marker gene tEGFR. **(C)** PTK7-CAR expression on transduced human CD4^+^ and CD8^+^ T cells was approximately 40–60% and 20–30%, respectively, as determined by tEGFR and CAR co-staining.

To assess the suitability of PTK7 as a target for CAR T cells, we designed three CARs (PTK7-CAR1, PTK7-CAR2, and PTK7-CAR3) each containing ascFv derived from one of three humanized antihuman PTK7 monoclonal antibodies (**Figure 1A**). The PTK7-specific scFv was fused to CD8α hinge and transmembrane domain with intracellular 4-1BB (CD137) co-stimulatory and CD3ζ activating signaling domains in tandem. To facilitate the detection of transduced T cells, a truncated EGFR (tEGFR) tag was included *via* T2A ribosomal skipping sequence. Expression of tEGFR alone served as a negative control. We synthesized full-length DNA encoding each of the CARs and cloned into a self-inactivating lentiviral vector. Then 293T cells were infected with CAR-encoding replication-incompetent lentiviruses where CAR and tEGFR displayed a linear co-expression pattern, indicating that tEGFR is a reliable marker for PTK7-CAR expression (**Figure 1B**). PBMC from healthy donors were then transduced with the lentiviruses following anti-CD3/CD28 bead stimulation, and both CAR and tEGFR expressions were determined by FACS analysis 5–7 days after transduction. We observed a similar linear co-expression pattern of both CAR and tEGFR in each CAR-transduced T cells with CAR transduction efficiency approximately 40–60% and 20–30% in CD4^+^ and CD8^+^ T cells, respectively (**Figures 1C** and **S1A**). Although there was a similar CAR expression positivity among three PTK7-CAR candidates, we consistently observed a high CAR expression per cell in the PTK7-CAR2 T cells (**Figure S1B**). Phenotypic analysis showed that PTK7-CAR T cells contained central memory, effector memory, and T stem cell memory, without significant differences among three candidates (**Figure S1C**). In addition, no difference in T-cell expansion without antigen stimulation was seen *in vitro* among control and those candidates (**Figure S1D**).

### PTK7-CAR T Cells Secrete Effector Cytokines and Proliferate After Exposure to PTK7-Expressingtumor Cells

**Figure 2:**
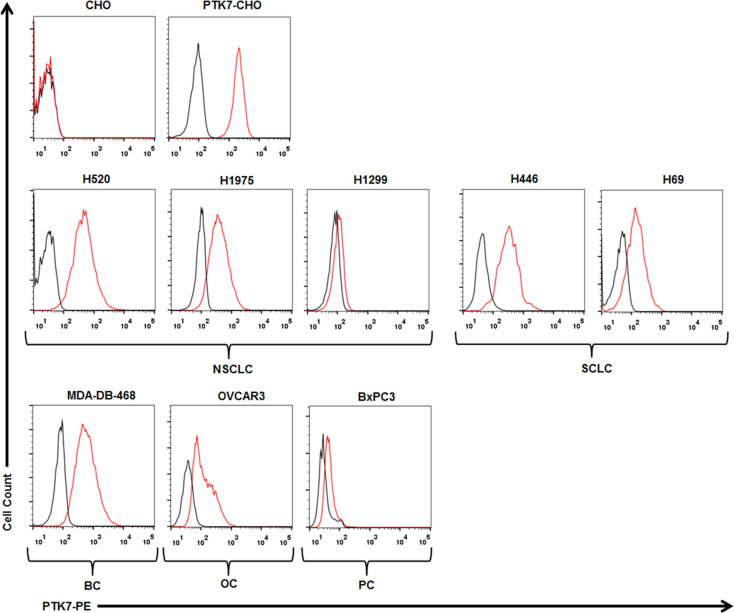
PTK7 is overexpressed on several tumor cell lines. CHO and PTK7-CHO cells served as negative and positive controls, respectively. PTK7 overexpression was observed on NSCLC (H520, H1975, H1299), SCLC (H446, H69), MDA-DB-468 breast cancer (BC), BxPC3 pancreatic cancer (PC), OVCAR3 ovarian cancer (OC) cells. Black and red lines denote the control (secondary antibody alone) and PTK7 staining, respectively.

**Figure 3:**
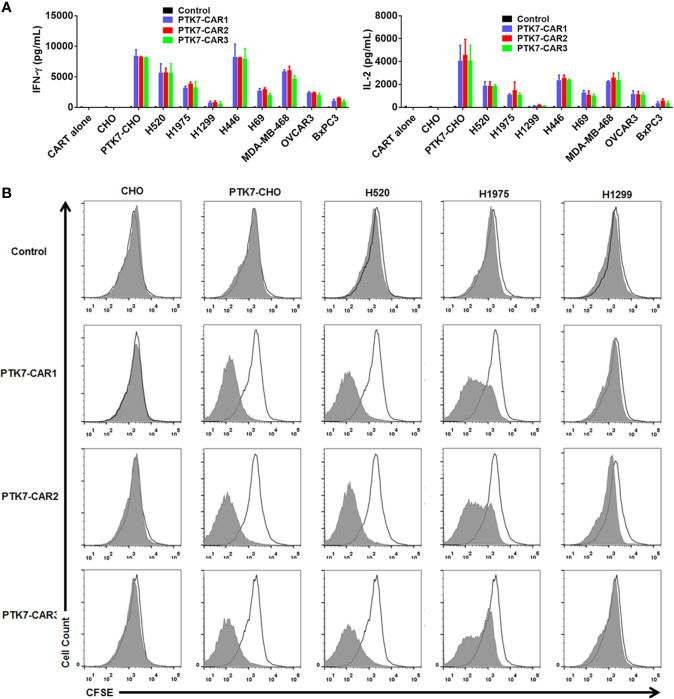
PTK7-CAR T cells release IFN-γ and IL-2 and proliferate in response to PTK7-positive target cells. **(A)** Control or PTK7-CAR T cells from healthy donors (n = 3) were co-cultured with CHO and PTK7-CHO and various PTK7-expressing tumor cell lines for 24 h before performing IFN-γ and IL-2 ELISA. Mean and SEM are shown. **(B)** T cells were labeled with CFSE and co-cultured for 3 days with CHO, PTK7-CHO, H520, H1975, or H1299 cells in the absence of exogenous IL-2, and CFSE dilution was analyzed by flow cytometry. A representative histogram from three independent assays is shown.

To test specific recognition by PTK7-CART cells, we initially exploited PTK7-negative parental CHO cells and stably transfected PTK7-expressing PTK7-CHO cells (**Figure 2**). PTK7-CAR T cells and control T cells of three donors were co-cultured with CHO or PTK7-CHO cells, and effector cytokine IFN-γ and IL-2 release in the supernatants were evaluated after 24 h (**Figure 3A**). PTK7-CAR T cells secreted significant amounts of IFN-γ and IL-2 after exposure to PTK7-CHO cells compared with control T cells; however, parental CHO cells did not stimulate PTK7-CAR T cells to produce effector cytokines, indicating that cytokine production requires both the expression of PTK7 on target cells and PTK7-CAR expression on transduced T cells. We confirmed the above findings using a panel of tumor cell lines naturally expressing the varying levels of PTK7 representative of multiple cancer types, including NSCLC (H520, H1975, H1299), SCLC (H446, H69), pancreatic (BxPC3), breast (MDA-DB-468), and ovarian (OVCAR3) cancer (**Figure 2**). Similarly, PTK7-CAR T cells produced a large amount of IFN-γ and IL-2, which is positively associated with the expression level of PTK7 on respective tumor cells (**Figure 3A**). Notably, PTK7-CAR2 T cells had a trend of producing a higher level of cytokines especially responding to stimulation by tumor cell lines expressing lower level of PTK7 (H1299 and BxPC3 cells), consistent with the higher level of CAR expression per cell in this construct (**Figure S1B**).

We also evaluated the antigen-specific proliferation of PTK7-CAR T cells in response to PTK7-expressing cells. T-cell proliferation was dependent on the expression level of PTK7 on target cells, and tumor cells with a high level of PTK7 expression induced more vigorous T-cell proliferation than that with a lower level of PTK7 expression (**Figure 3B**). Again, PTK7-CAR2 T cells exhibited a trend of more potent proliferation when stimulated with tumor cells expressing a lower level of PTK7.

### PTK7-CAR T Cells Specifically Kill PTK7-Expressing Tumor Cells and Retain Effector Function Upon Recursive Target Exposure

**Figure 4:**
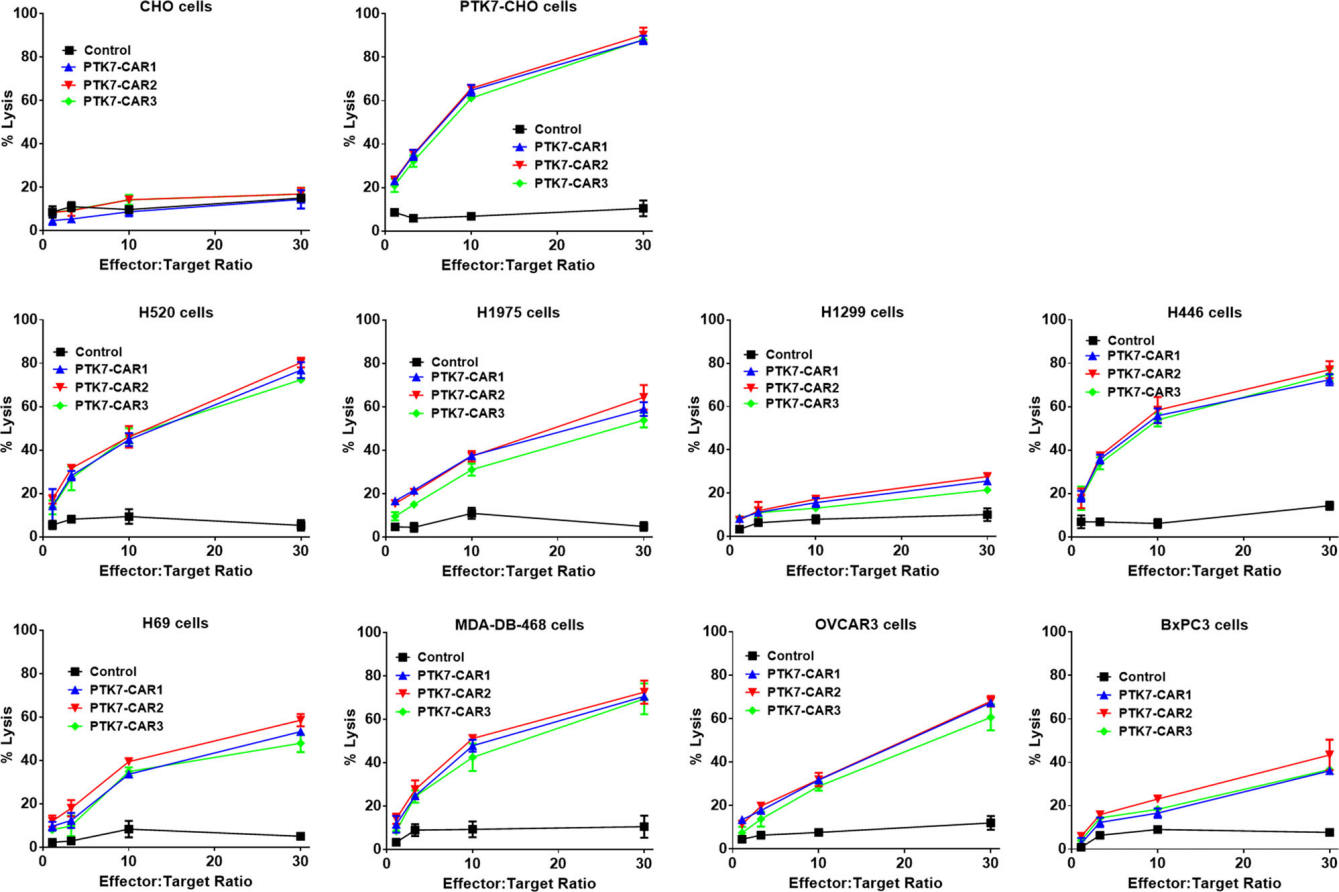
PTK7-CAR T cells kill PTK7-positive tumor cell lines. GL-expressing tumor target cells were co-cultured with control or PTK7-CAR T cells at the varying effector-to-target ratios in triplicate wells of white 96-well plates. Target cell viability was monitored 18 h later by using Bright-Glo™ Luciferase Assay System according to the manufacturer’s instructions. The percent lysis (%) was calculated by using the following equation: 1-[bioluminescence value in sample well (target cells + CAR T cells)/maximum bioluminescence value (target cells alone)]. Shown are means ± SEM of % cell killing in triplicate wells.

**Figure 5:**
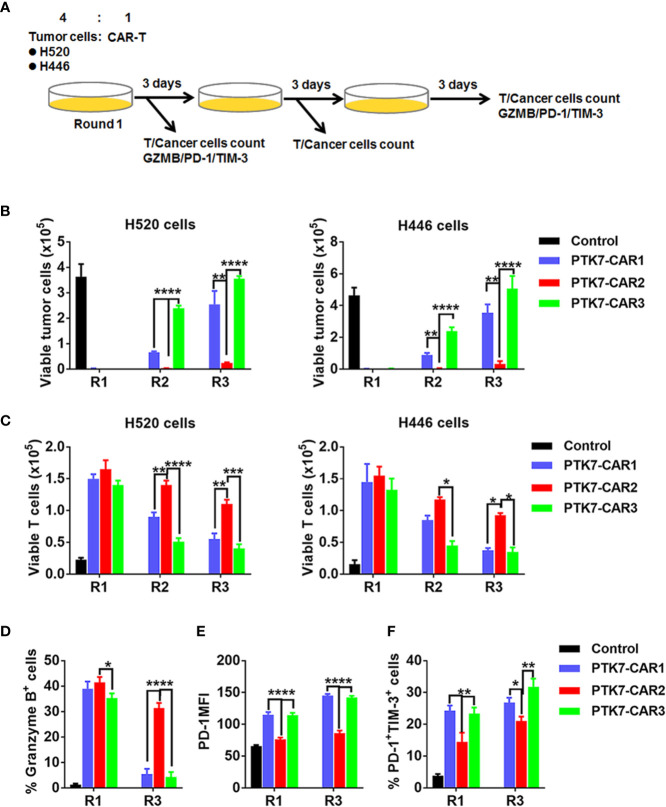
PTK7-CAR2 T cells retain effector function upon recursive target exposure. **(A)** Schematics of the long-term cytotoxicity assay. **(B)** Counts of H520 and H446 target cells after each round of recursive co-culture (rounds 1–3, R1–R3) with control or PTK7-CAR T cells. **(C)** Counts of control or PTK7-CAR T cells after each round of recursive co-culture with target cells. **(D)** Intracellular staining for granzyme B of control or PTK7-CAR T cells at the end of round 1 and 3 co-culture with H520 tumor cells. **(E)** PD-1 expression in control or PTK7-CAR T cells after rounds 1 and 3 of recursive co-culture with H520 tumor cells. **(F)** Percentage of PD-1^+^TIM-3^+^ cells in control or PTK7-CAR T cells after rounds 1 and 3 of recursive co-culture with H520 tumor cells. Data are shown as mean ± SEM (n = 3). *P < 0.05, **P < 0.01, ***P < 0.001, and ****P < 0.0001, determined by repeated-measures two-way ANOVA with Tukey’s *post hoc* test.

We next evaluated the specific killing of PTK7-positive tumor cells by PTK7-CAR T cells in both short-term (18 h) and recursive long-term (three rounds with each round of 3 days) cytotoxicity assays. In the short-term assays, PTK7-CAR T cells exhibited a robust dose-dependent cytotoxicity against PTK7-expressing PTK7-CHO cells and tumor cells but not parental CHO cells (**Figure 4**). Noticeably, PTK7-CAR2 T cells demonstrated a comparatively higher degree of cytotoxicity against tumor cells expressing the lower level of PTK7 (H69, BxPC3, and H1299 cells). As PTK7 has be reported to be shed from tumor cells in a soluble form (12), we also evaluated the effect of soluble PTK7 on the cytotoxicity of PTK7-CAR T cells, which showed it minimally impacted the tumor killing of these cells (**Figure S2**). Maintenance of specific cytotoxicity and proliferative response exposure to continuous antigen stimulation has been described to be associated with preferential antitumor activity (30, 31). To mimic that context *in vitro*, we performed the recursive long-term cytotoxicity assay where CAR T cells were exposed to recursive target cells at a certain ratio, and tumor cell killing and T cell proliferation served as readouts after each round (**Figure 5A**). We observed that PTK7-CAR2 T cells retained effective through three rounds of tumor challenge, whereas the other two PTK7-CAR T cells failed to control tumor cell growth after the first or second round of challenge (**Figure 5B**). In parallel, PTK7-CAR2 T cells had a better persistence after each round of challenge (**Figure 5C**). PTK7-CAR2 T cells also exhibited superior effector function at the individual cell level as evidenced by higher levels of lytic enzyme granzyme B expression and reduced expression of the exhaustion markers PD-1 and lower percentage of PD-1^+^TIM-3^+^ cells as compared to the other two PTK7-CAR T cells (**Figures 5D–F** and **S3**).

### PTK7-CAR T Cells Mediate Antitumor Activity Against Established Lung Cancer Xenografts

**Figure 6:**
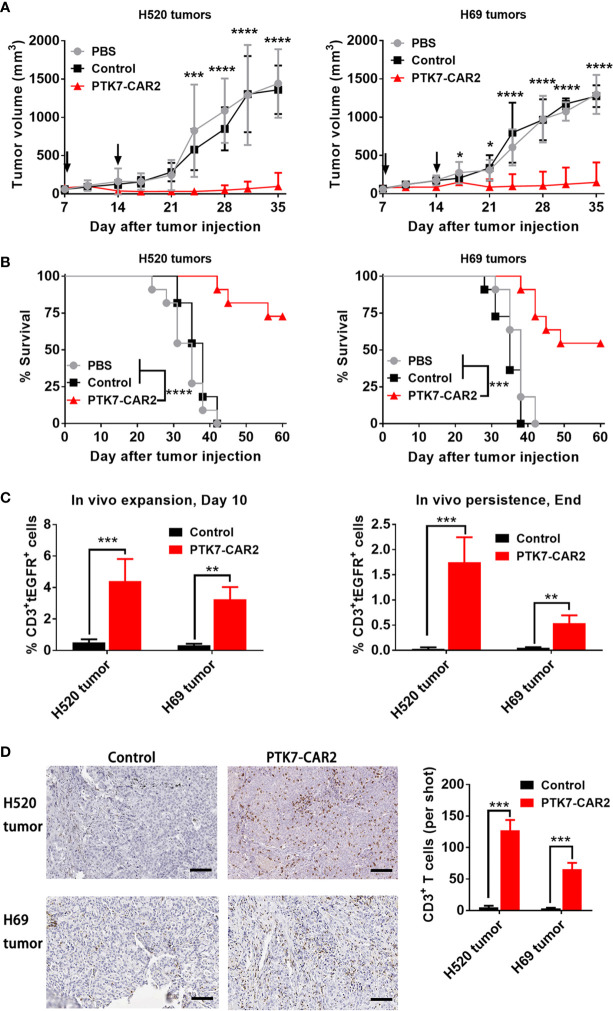
Systemic treatment with PTK7-CAR2 T cells leads to tumor growth control and increased survival of mice in both human tumor xenograft models. **(A)** NSG mice were s.c. implanted with H520 or H69 tumor cells, after 7 days, received two intravenous infusion of control or PTK7-CAR2 T cells (5 × 10^6^ cells in 100 µl PBS) week apart and tumor growth quantified by measuring tumor size. Data are shown as mean ± SEM (n = 5 mice per group). *P < 0.05, ***P < 0.001, and ****P < 0.0001, determined by repeated-measures two-way ANOVA with Tukey’s *post hoc* test. **(B)** Kaplan–Meier survival curves summarizing three independent experiments (n = 11 mice per group). ***P < 0.001, and ****P < 0.0001 determined by log-rank test. **(C)** Frequency of human CD3^+^tEGFR^+^ CAR T cells in the peripheral blood collected 10 days after T cell infusion or at the end of experiment. Data are shown as mean ± SEM (n = 4 mice per group). **P < 0.01 and ***P < 0.001, determined by repeated-measures two-way ANOVA with Tukey’s *post hoc* test. **(D)** Representative IHC images and quantification of T-cell infiltration in tumor tissues (n = 3) from treated mice harvested at the end of experiment. Scale bars, 100 µm. Data are shown as mean ± SEM (n = 3 mice per group). ***P < 0.001, determined by repeated-measures two-way ANOVA with Tukey’s *post hoc* test.

In view of the *in vitro* preferential target-specific recognition and cytotoxicity of PTK7-CAR2 T cells as well as the fact that the antibody from which the scFv used by PTK7-CAR2 is derived has been tested in the clinical trial (12), we evaluated the *in vivo* antitumor activity of these candidate CAR T cells in the xenograft tumor models established from two lung cancer cell lines with distinct antigen expression: H520 and H69 cells with high or moderate level of PTK7 expression respectively as determined by flow cytometry and IHC staining of cell line-derived xenografts (**Figures 2** and **S4**). NSG mice (n = 3–5/group) were s.c. inoculated with H520 or H69 tumor cells. Seven days later mice started to receive two injection of control or PTK7-CAR2 T cells 1 week apart, and tumor growth was monitored by measuring tumor size. Three independent experiments with T cells from different donors showed that administration of PTK7-CAR2 T cells greatly inhibited tumor growth and significantly prolonged the overall survival of mice bearing H520 (p<0.0001) and H69(p<0.001) tumors (**Figures 6A, B**), culminating in tumor-free survival of more than half of mice at the end of experiment in both tumor models. In contrast, mice treated with control T cells or PBS developed a rapidly progressive tumor, necessitating euthanasia approximately 6 weeks after tumor inoculation, excluding the contribution of allogeneic reactivity to antitumor effect of PTK7-CAR T cells. Accordingly, PTK7-CAR2 T cells exhibited superior initial expansion (day 10 after T-cell infusion) *in vivo* in the peripheral blood and extended persistence when mice were sacrificed (**Figures 6C** and **S5**). In addition, CD3^+^ T-cell infiltration in tumor xenografts was determined by IHC staining at the endpoint of the experiment, and mice treated with PTK7-CAR2 T cells exhibited a prominent accumulation of T cells within tumor tissues compared to mice treated with control T cells (**Figure 6D**).

Importantly, there was no overt evidence of adverse reaction associated with the infusion of PTK7-CAR2 T cells to mice, as measured by body weight loss and physical signs of toxicity in above animal studies performed (**Figure S6**).

### PTK7-CAR2 T Cells Do Not Mediate Detectable On-Target Off-Tumor Toxicity

**Figure 7:**
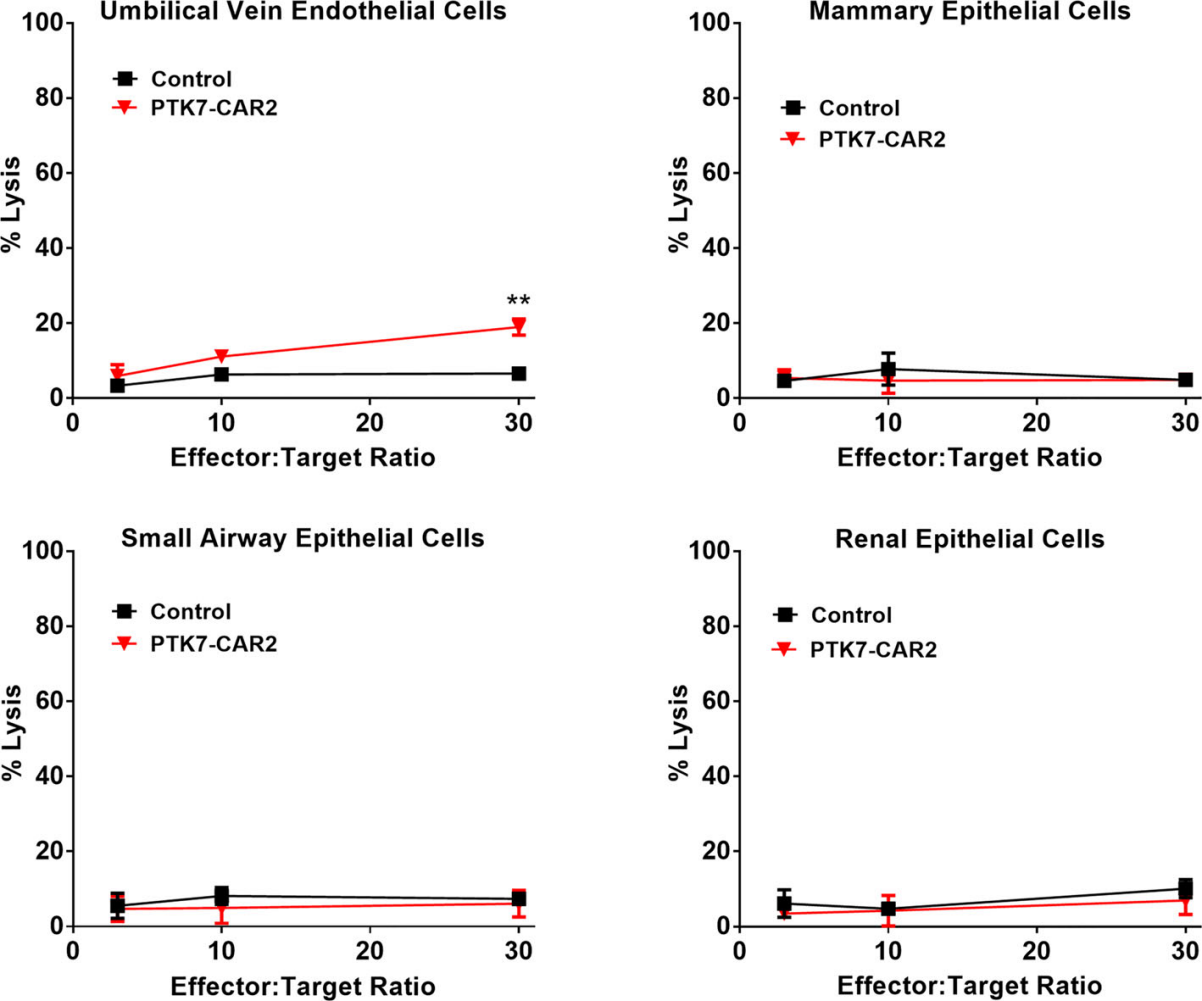
PTK7-CAR2 T cells do not mediate detectable on-target off-tumor toxicity. Control or PTK7-CAR T cells were tested reactivity against a panel of primary human normal epithelial cells or HUVECs in the cytotoxicity assays at the indicated effector-to-target ratios. Shown are mean ± SEM of % cell killing in triplicate wells. **P < 0.01, determined by repeated-measures two-way ANOVA with Tukey’s *post hoc* test.

A previous study shows that a low level of PTK7 expression can be detected in the normal epithelial cells from some tissues, including mammary gland, lung, kidney, esophagus, and urinary bladder. We assessed the expression of PTK7 in the normal human TMA using the rabbit polyclonal anti-PTK7 antibody. Slides were analyzed blindly by an experienced pathologist. Major organs such as heart, brain, lung, liver, and spleen are PTK7 negative, while focal, weak to moderate PTK7-positive staining was observed in the cytoplasm of some normal human tissues (**Figure S7** and **Table S1**). The highest expression was observed in the stomach with moderate to strong cytoplasmic staining of gastric epithelium, colon with weak cytoplasmic staining of epithelium, and kidney with weak cytoplasmic staining of tubule epithelial cells. Since on-target off-tumor toxicity is a key limiting factor when developing novel CAR T therapies, we roughly address this concern using a panel of primary human normal cell lines with low-level expression of PTK7 (**Figure S8**). Control or PTK7-CAR2 T cells were co-cultured with the primary human normal epithelial cell lines from the mammary gland (Mammary Epithelial Cells, MECs), lung (Small Airway Epithelial Cells, SAECs), and kidney (Renal Epithelial Cells, RECs) and human umbilical vein endothelial cells (HUVECs), and cytotoxicity assays were performed. Compared to control T cells, PTK7-CAR2 T cells did not exhibit more potent killing against this limited panel of normal human primary cells, except for low-level cytotoxicity of HUVECs that was only observed at the highest effector-to-target ratio tested (**Figure 7**). As not all human tissues with PTK7 expression are represented, these studies are limited but can serve as an initial screen for off-tumor activity.

## Discussion

Here, we described the generation and antitumor efficacy of second-generation PTK7-targeting CAR T cells with 4-1BB intracellular co-stimulatory signaling domain and demonstrated antigen-specific cytokine production and cytotoxicity against multiple PTK7-positive tool cells and naturally expressing human tumor cells *in vitro*; more importantly, *in vitro* recursive tumor challenge assays pointed to a preferred candidate (PTK7-CAR2) out of three CAR constructs in terms of repetitive target cell killing, CAR T-cell expansion and exhaustion–associated phenotypes, which was previously reported to be associated with *in vivo* antitumor effect of CAR T cells (32). Using *in vivo* lung cancer cell line–derived xenograft models, we showed that PTK7-CAR2 T cells significantly inhibited tumor growth and prolonged overall survival of tumor-bearing mice. The reason why PTK7-CAR2 exhibited a better functionality remains to be explored; however, we consistently observed a high CAR expression per cell in the PTK7-CAR2 T cells (**Figure S1B**), which may underlie the fact that PTK7-CAR2 T cells had a better response to stimulation by tumor cell lines expressing lower levels of PTK7. It is possible that the unique scFv sequence in this construct makes this CAR more easy expression or more stable on the CAR-T cell surface, enabling CAR-T cells a better response in recursive target exposure. We did not evaluate PTK7-CARs integrating CD28 co-stimulation as CD28-containing CAR T cells have undesirable increases in T cell exhaustion markers, limited persistence, and increased possibility of recognizing normal cells with very low levels of antigen as previously reported (30, 33). Although further studies will be needed to evaluate the antitumor efficacy of PTK7-CAR T cells in a more clinically relevant setting such as using PDXs and patient-derived cancer cell lines, our data support PTK7-CAR T cells as a viable therapeutic option for lung cancers and many other solid cancers with PTK7 overexpression given that it is impractical to develop blocking antibodies or small-molecule inhibitors as typically done with receptor tyrosine kinases due to PTK7’s lack of catalytic activity.

Several reports have identified PTK7 as a potential antigen target in solid tumors. Previous studies documented that PTK7 is overexpressed in multiple types of solid cancer, and more significantly, its expression is enriched in TICs/CSCs from PDXs or cancer cell lines. As TICs/CSCs with unlimited self-renewal capacity and differentiation potential have been broadly considered to be source to tumor recurrence, metastasis, and therapeutic resistance, it is reasonable to hypothesize that a durable antitumor efficacy would be achieved if specifically targeting TIC/CSCs by immunotherapeutic modalities, including CAR T-cell therapy. In fact, CAR T cells targeting several biomarkers of TICs/CSCs, including CD133, CD24, Receptor tyrosine kinase-like orphan receptor 1 (ROR1), or the epithelial cell adhesion molecule (EpCAM), have been developed and exhibited the excellent antitumor effects in preclinical models (34–40); more importantly, CD133-targeting CAR T cells alone or in combination have demonstrated antitumor activity in treating patients with CD133-postive metastatic malignancies with controllable toxicities in clinical trials (41, 42). Intriguingly, both PTK7 and ROR1 belong to Wnt ligand binding receptors with important roles in the non-canonical Wnt signaling (10). ROR1 exhibits high and homogeneous cell surface expression in many epithelial tumors with expression profile similar to PTK7, and targeting ROR1 with CAR T-cell therapy improved survival in xenograft models of ROR1^+^ human tumors with treating lung and breast cancer in an ongoing clinical trial (NCT02706392) (38, 40). Thus, our result documenting a potent antitumor effect of PTK7-CAR T cells adds PTK7 to the kind list of ROR1, which, as a member of Wnt signaling-related pseudokinases, had a characteristic enriched expression in TIC/CSCs and is suitable as a potential therapeutic target for cancer immunotherapy.

On-target off-tumor effect is a major concern when developing CAR T-cell therapy targeting less tolerable TAAs for solid tumors (8). On-target toxicities have been observed in clinical trials with CAR T cells specific for antigens that are shared on some normal tissues (43, 44), and a critical issue to be addressed is whether targeting PTK7 will be safe. Damelin et al. have shown that PTK7 is absent in vital organs, but they detected a low level of expression in esophagus, urinary bladder, kidney, mammary gland, lung, ovary, uterus, and digestive tract with more prominent expression in stromal part (12); in addition, previous studies detected PTK7 expression in human hematopoietic progenitors committed to myeloid and T lymphoid lineages, illustrating the potential for toxicity to normal cells (45–48). Roughly consistent with these previous studies, we also did not detect PTK7 expression in normal human major organs; however, focal, weak to moderate PTK7-positive staining was observed in the cytoplasm of some normal human tissues, including digestive tracts (stomach, esophagus, colon) and kidney. We further evaluated the activity of PTK7-CAR T cells *in vitro* against a normal cell panel that included mammary, lung, kidney epithelial cells, and HUVECs where a low level of lysis against HUVECs was detected only at the highest effector-to-target ratio tested, which is consistent with comparatively higher PTK7 expression on these cells as determined by FACS. As CAR-T cells will first accumulate in the lung through blood vessels, a very high local concentration may be achieved when CAR-T cells are intravenously infused, leading to the blood vessel in the lung attacked by CAR-T cells and consequently on-target off-tumor toxicity. In addition, if applied for lung cancer treatment, a high level of cytokines released from the on-target on-tumor recognition may constitute another important concern as this may induce pulmonary edema, which may be lethal if not diagnosed and treated timely. Although mouse and human PTK7 protein has 90.93% homology in total sequence with 92.98% homology in the extracellular domain, which means that tumor-bearing mouse model should be suitable for the evaluation of on-target toxicity, however, we cannot evaluate the potential toxicity profiles of PTK-CAR T cells in current murine tumor models due to lack of cross-reactivity with murine counterpart of humanized antihuman PTK7 antibodies used to construct scFv part of our PTK-CARs. Positively, Damelin et al. showed that a PTK7-targeting ADC did not exhibit target-dependent toxicity in any of the tissues examined, including those with PTK7 expression (12). As the scFv we used to generate PTK7-CAR2 is derived from the same antibody (Hu24) of that PTK7-ADC, the non-clinical safety profile of PTK7-ADC in that study provides some evidence of safety and potential toxicity estimate of PTK7-CAR2 T cells *in vivo*. Caution still should be taken when translating this PTK7-CAR T cells into clinic considering different mechanisms of action and target recognition sensitivity (potency) between ADC and CAR T cells directing the same targets. In this regard, CAR-T cells targeting epithelial cell adhesion molecule (EpCAM), a tumor-associated antigen overtly presented on the cell surface of various carcinomas, is a typical precedent. Although anti-human EpCAM CAR-T cells unable of recognizing mouse EpCAM eradicated established tumor xenografts without toxicities in the immunodeficient animal models, anti-mouse EpCAM CAR-T cells induced severe pulmonary immunopathology in theimmunocompetent mice due to CAR-T recognition of basal EpCAM expression in normal lung (49). In addition, we may learn from the experience of targeting ROR1 by CAR T cells as these two molecules have similar expression profiles in both normal and tumor tissues (10, 50). Although ROR1-CAR T cells (derived from R12- and 2A-scFv) without cross-reactivity with murine ROR1 exhibited no evident toxicity in NSG mice tumor model, murine ROR1-specific CAR T cells (derived from R11-scFv) induced lethal bone marrow failure due to recognition of ROR1^+^ stromal cells, which can be rescued by the logic-Gated strategy of CAR construction (38, 40). Thus, the same configuration should be considered when similar results are seen in future investigations of PTK7-CAR T-cell’s toxicity profiles; alternatively, tuning scFv affinity and/or concomitantly integrating different co-stimulatory domains may ameliorate the potential concern of on-target off-tumor effect as typical representation for CAR T-cell therapy targeting a range of different antigens including but not limited to HER2, EGFR, CD38 (8, 51, 52). Given the above inherent risks, multiple inducible safety controls that can be built into or applied in conjunction with CAR-T cells should be considered, such as inducible caspase 9 (iCasp9) or tEGFR tag as we integrated in CAR design where tEGFR-expressing CAR-T can be depleted by commercially available antibody cetuximab in case of emergent side effects (53). In sum, further thorough investigations are definitely needed to fully explore the potential toxicities of PTK7-CAR T cells before translating into clinic by using PTK7-CARs with cross-reactivity in mouse and even non-human primate models.

## Conclusion

Here we describe a PTK7 targeting strategy that is based upon CAR T-cell engineering. This synthetic biology approach overcomes the issues related with PTK7 being pseudokinase unsuited for developing antibody and small-molecule inhibitors as therapeutic agents, and is supported by the effector functions of modified T-cell in order to deliver PTK7-specific cytotoxicity. In summary, the data presented herein serve as an initial step for future clinical development of PTK7-CAR T-cell therapy safely and efficiently treating PTK7-expressing lung cancer and other malignancies.

## Data Availability Statement

The original contributions presented in the study are included in the article/**Supplementary Material**. Further inquiries can be directed to the corresponding author.

## Ethics Statement

This study was approved by ethical committees of the Harbin Medical University Cancer Hospital.

## Author Contributions

Conception and design of studies: YJ, GR, HW, AG. Acquisition, analysis and interpretation: YJ, GL, LF, YL, ME, LW, XL, YYL, YWL, HW, AG. Drafting article: YJ, AG. Critical review and discussion: GR, HW. All authors contributed to the article and approved the submitted version.

## Funding

This work was supported by the grants from the National Science and Technology Major Project (2017ZX10203206), the National Natural Science Foundation of China (81572461, 81672274, and 81372528), the Science Foundation of the Postdoctoral Department of Heilongjiang Province (No. LBH-Z16114), and the Harbin Medical University Science Foundation of Innovative Science Research (2017LCZX107).

## Conflict of Interest

The authors declare that the research was conducted in the absence of any commercial or financial relationships that could be construed as a potential conflict of interest.

## Publisher’s Note

All claims expressed in this article are solely those of the authors and do not necessarily represent those of their affiliated organizations, or those of the publisher, the editors and the reviewers. Any product that may be evaluated in this article, or claim that may be made by its manufacturer, is not guaranteed or endorsed by the publisher.

## Supplementary Material

Supplementary Figure 1 **(A)** The percentage of CAR expression on T cells transduced with control or PTK-CAR lentiviruses. Data are shown as mean ± SEM (n = 5 donors). **(B)** The intensity of CAR expression in both CD4 and CD8 T cells from 3 different CAR constructs. **(C)** CAR T-cell memory phenotypic analysis in both CD4+ and CD8+ cells based on CD45RO and CCR7 expression as follows: Tn/Tscm naive/stem cell memory (CD45RO-/CCR7+), Tem central memory (CD45RO+/CCR7+), Tem effector memory (CD45RO+/CCR7-), Teff effector cells (CD45RO-/CCR7-) (n = 5 donors). **(D)** The in vitro expansion curve of control or PTK-CAR T cells. Data are normalized on the starting input T cell number and shown as mean ± SEM of triplicates from one representative donor. *P < 0.05, ***P < 0.001, determined by two-way ANOVA with Turkey’s post hoc test.

Supplementary Figure 2 Soluble PTK7 antigen does not impact tumor killing of PTK7-CAR T cells in vitro. PTK7-CAR T cell-mediated tumor killing of H520 **(A)** or H1975 **(B)** cells at the effector-to-target ratio of 10 in the presence or absence of 10 µg/mL of soluble purified PTK7 antigen in the short-term cytotoxicity assay.

Supplementary Figure 3 **(A)** Representative plots of flow cytometric analysis of intracellular granzyme B (GZMB) expression in control or PTK7-CAR T cells at the end of round 1 and 3 co-culture with H520 tumor cells. **(B)** Representative plots of flow cytometric analysis of PD-1 and TIM-3 expression on control or PTK7-CAR T cells at the end of round 1 and 3 co-culture with H520 tumor cells.

Supplementary Figure 4 Immunohistochemistry of PTK7 in tumor xenografts. Immunohistochemistry with polyclonal anti-PTK7 antibody was performed on formalin-fixed, processed, and paraffin-embedded (FFPE) tumor tissues from H520 **(A)** or H69 **(B)** xenografts. Scale bar, 100 μm.

Supplementary Figure 5 Representative plots of flow cytometric analysis of human CD3+tEGFR+ CAR T cells in the peripheral blood collected 10 days (D10) after T cell infusion or at the end of experiment (End) from the H520 **(A)** or H69 **(B)** xenograft tumor models.

Supplementary Figure 6 Body weights were measured before tumor injection, before CAR T-cell injection, and 7 and 28 days after CAR T-cell injection and compared with PBS or control T-cell treated NSG mice. Lines indicate means ± SEM (n = 5 mice).

Supplementary Figure 7 Representative micrographs of PTK7 expression in indicated normal human organs assessed by staining with the rabbit polyclonal anti-PTK7 antibody (Invitrogen) at the final concentration of 1 µg/ml. Micrographs are representative of at least 2-3 sections per tissue. Magnification, x 40.

Supplementary Figure 8 PTK7 expression on the primary human normal epithelial cell lines from mammary gland (Mammary Epithelial Cells, MECs), lung (Small Airway Epithelial Cells, SAECs), and kidney (Renal Epithelial Cells, RECs) and human umbilical vein endothelial cells (HUVECs) determined by FACS analysis. Black and red line denote the control (secondary antibody alone) and PTK7 staining respectively.

## Abbreviations

CAR, chimeric antigen receptor; CSCs, cancer stem cells; scFv, single-chain variable fragment; ACT, adoptive cell therapy; TM, transmembrane; TCR, T cell receptor; MHC, major histocompatibility complex; HLA, Human Leucocyte Antigen; TAA, tumor-associated antigens; CCK-4, colon carcinoma kinase 4; RTKs, receptor tyrosine kinases; PCP, planar cell polarity; VEGF, vascular endothelial growth factor; TNBC, triple-negative breast cancer; NSCLC, non-small-cell lung cancer; OVCA, ovarian cancer; ESCC, cervical cancer, esophageal squamous cell carcinoma; HCC, hepatocellular carcinoma; TICs, enriched in tumor-initiating cells; PDXs, patient-derived xenografts; ADC, antibody-drug conjugate; FBS, fetal bovine serum; HUVECs, human umbilical vein endothelial cells; IHC, immunohistochemistry; tEGFR, truncated EGFR; ROR1, Receptor tyrosine kinase-like orphan receptor 1; EpCAM, epithelial cell adhesion molecule.
