# Supplementary figures and images for: PTK7-Targeting CAR T-Cells for the Treatment of Lung Cancer and Other Malignancies
Source: Front Immunol. 2021 Aug 12;12:665970. doi: 10.3389/fimmu.2021.665970 (PMC8406764; doi:10.3389/fimmu.2021.665970)

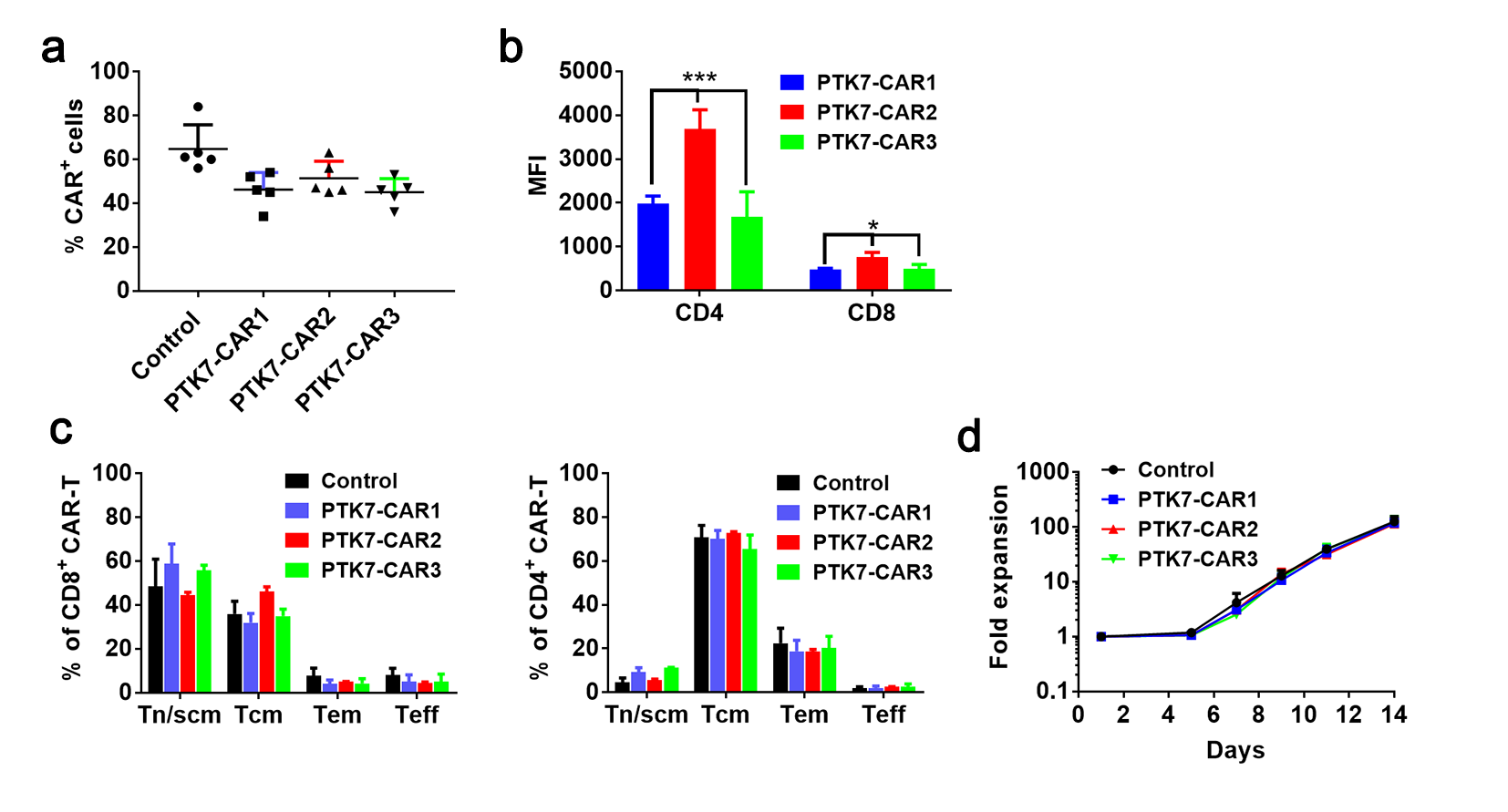

Supplement: Supplementary Figure 1 — (A) The percentage of CAR expression on T cells transduced with control or PTK-CAR lentiviruses. Data are shown as mean ± SEM (n = 5 donors). (B) The intensity of CAR expression in both CD4 and CD8 T cells from 3 different CAR constructs. (C) CAR T-cell memory phenotypic analysis in both CD4+ and CD8+ cells based on CD45RO and CCR7 expression as follows: Tn/Tscm naive/stem cell memory (CD45RO-/CCR7+), Tem central memory (CD45RO+/CCR7+), Tem effector memory (CD45RO+/CCR7-), Teff effector cells (CD45RO-/CCR7-) (n = 5 donors). (D) The in vitro expansion curve of control or PTK-CAR T cells. Data are normalized on the starting input T cell number and shown as mean ± SEM of triplicates from one representative donor. *P < 0.05, ***P < 0.001, determined by two-way ANOVA with Turkey’s post hoc test. [file Image_1.tif]

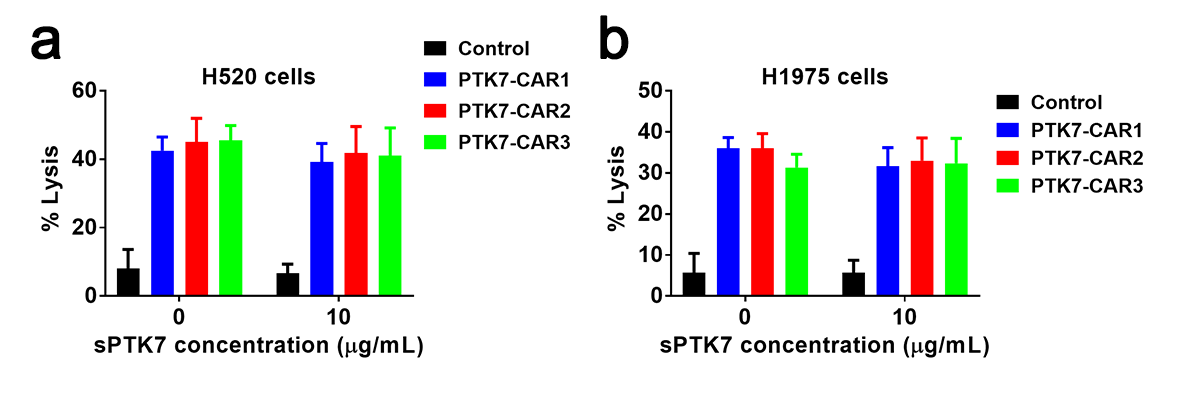

Supplement: Supplementary Figure 2 — Soluble PTK7 antigen does not impact tumor killing of PTK7-CAR T cells in vitro. PTK7-CAR T cell-mediated tumor killing of H520 (A) or H1975 (B) cells at the effector-to-target ratio of 10 in the presence or absence of 10 µg/mL of soluble purified PTK7 antigen in the short-term cytotoxicity assay. [file Image_2.tif]

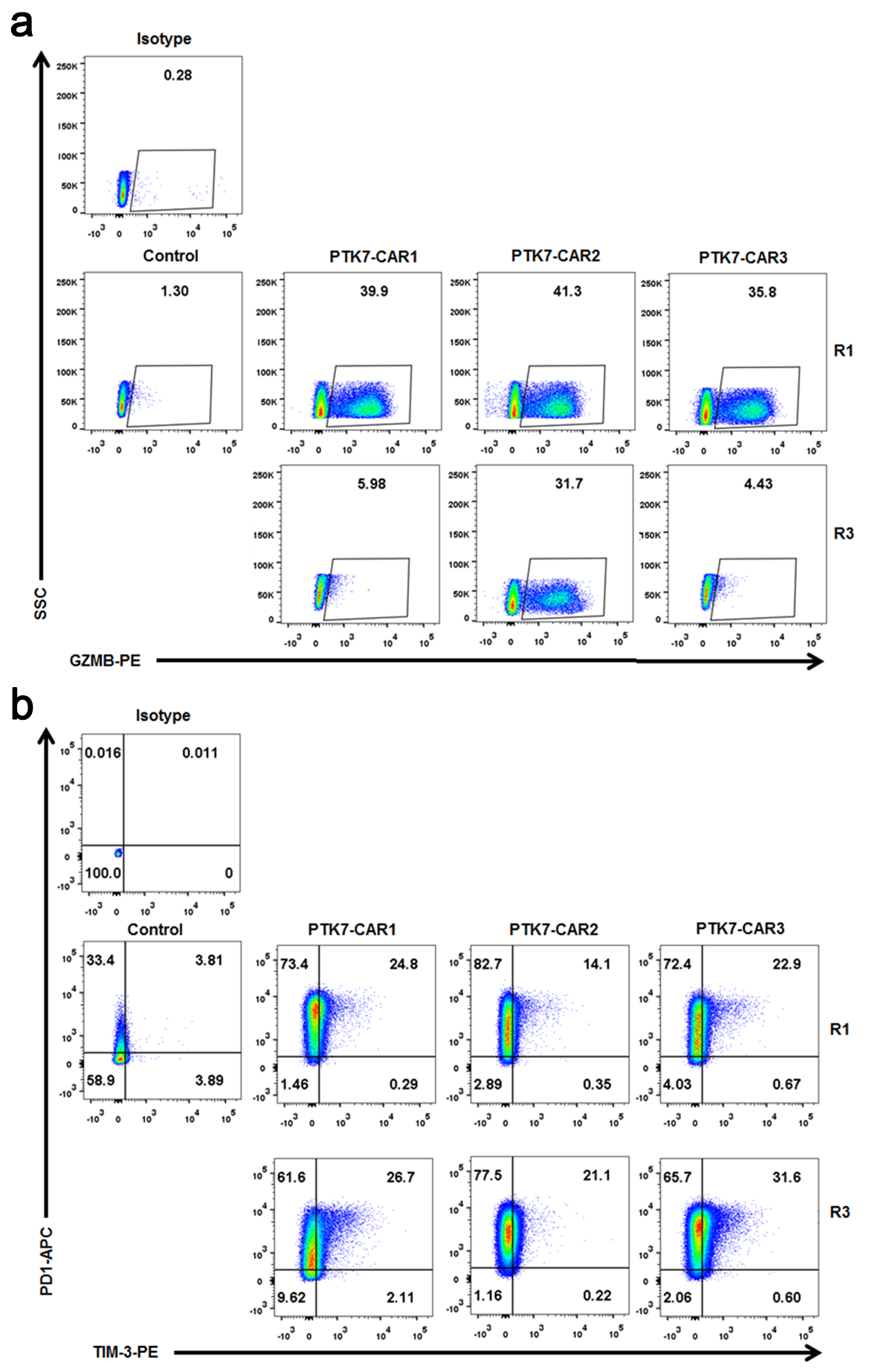

Supplement: Supplementary Figure 3 — (A) Representative plots of flow cytometric analysis of intracellular granzyme B (GZMB) expression in control or PTK7-CAR T cells at the end of round 1 and 3 co-culture with H520 tumor cells. (B) Representative plots of flow cytometric analysis of PD-1 and TIM-3 expression on control or PTK7-CAR T cells at the end of round 1 and 3 co-culture with H520 tumor cells. [file Image_3.tif]

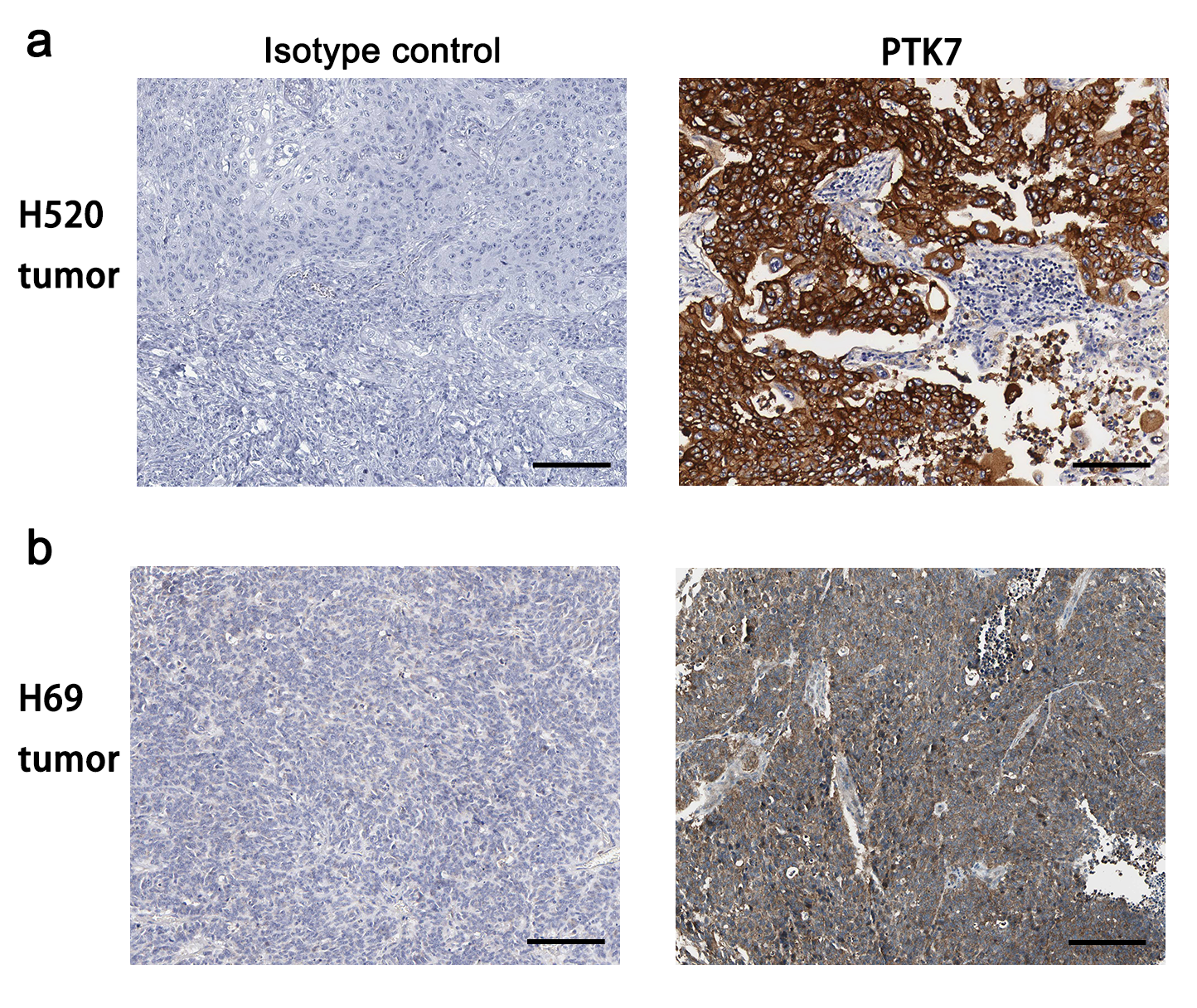

Supplement: Supplementary Figure 4 — Immunohistochemistry of PTK7 in tumor xenografts. Immunohistochemistry with polyclonal anti-PTK7 antibody was performed on formalin-fixed, processed, and paraffin-embedded (FFPE) tumor tissues from H520 (A) or H69 (B) xenografts. Scale bar, 100 μm. [file Image_4.tif]

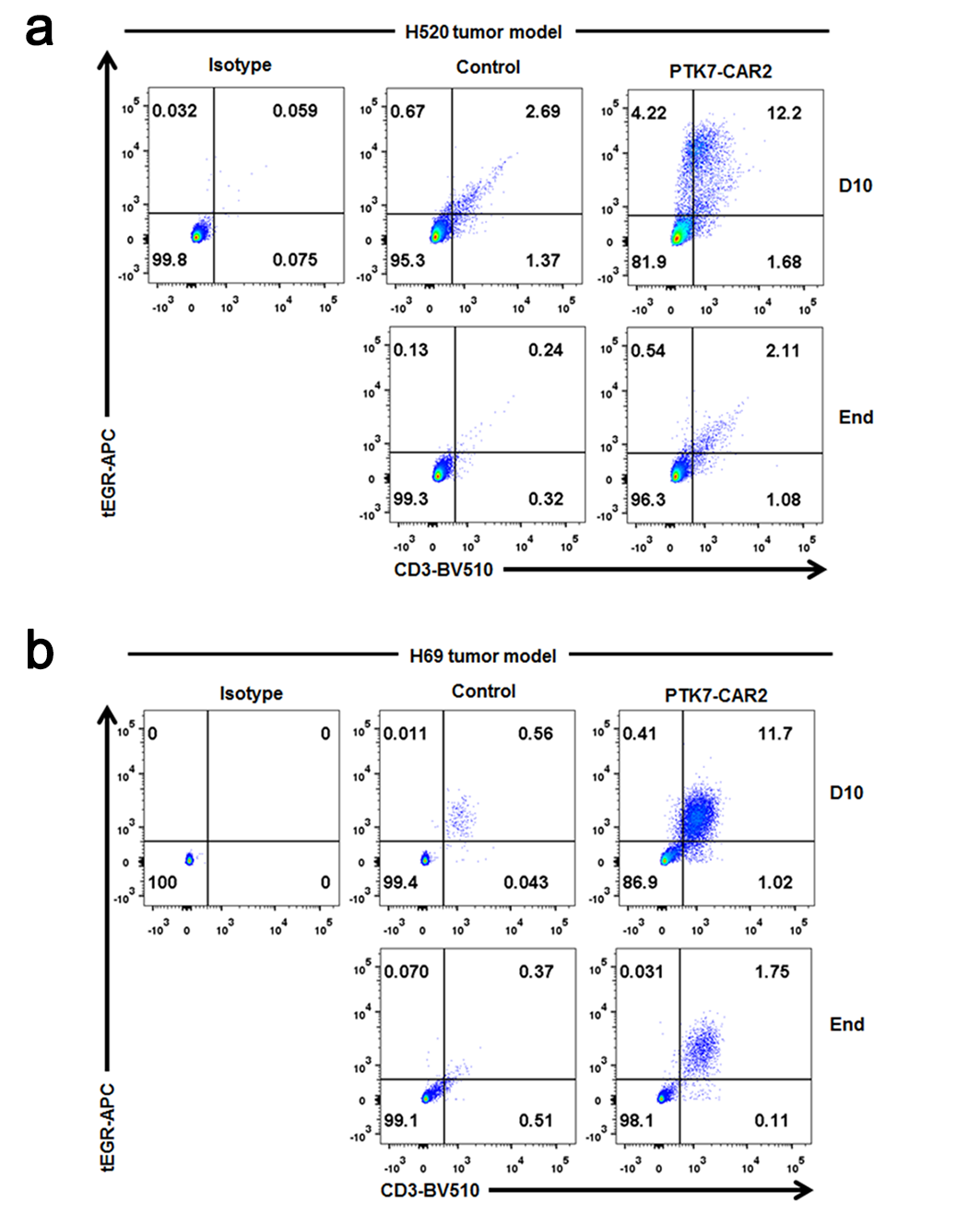

Supplement: Supplementary Figure 5 — Representative plots of flow cytometric analysis of human CD3+tEGFR+ CAR T cells in the peripheral blood collected 10 days (D10) after T cell infusion or at the end of experiment (End) from the H520 (A) or H69 (B) xenograft tumor models. [file Image_5.tif]

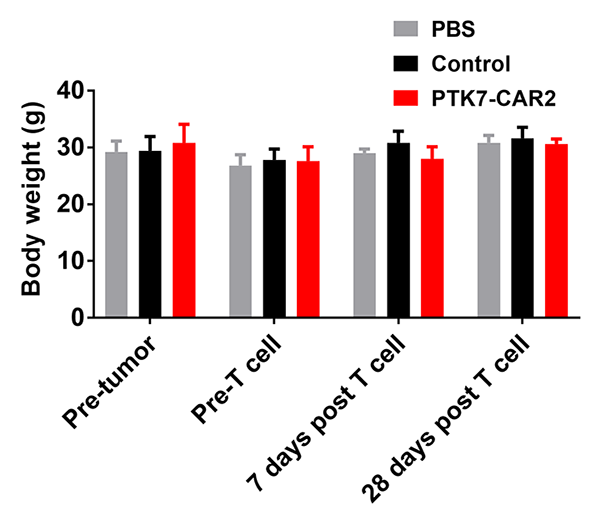

Supplement: Supplementary Figure 6 — Body weights were measured before tumor injection, before CAR T-cell injection, and 7 and 28 days after CAR T-cell injection and compared with PBS or control T-cell treated NSG mice. Lines indicate means ± SEM (n = 5 mice). [file Image_6.tif]

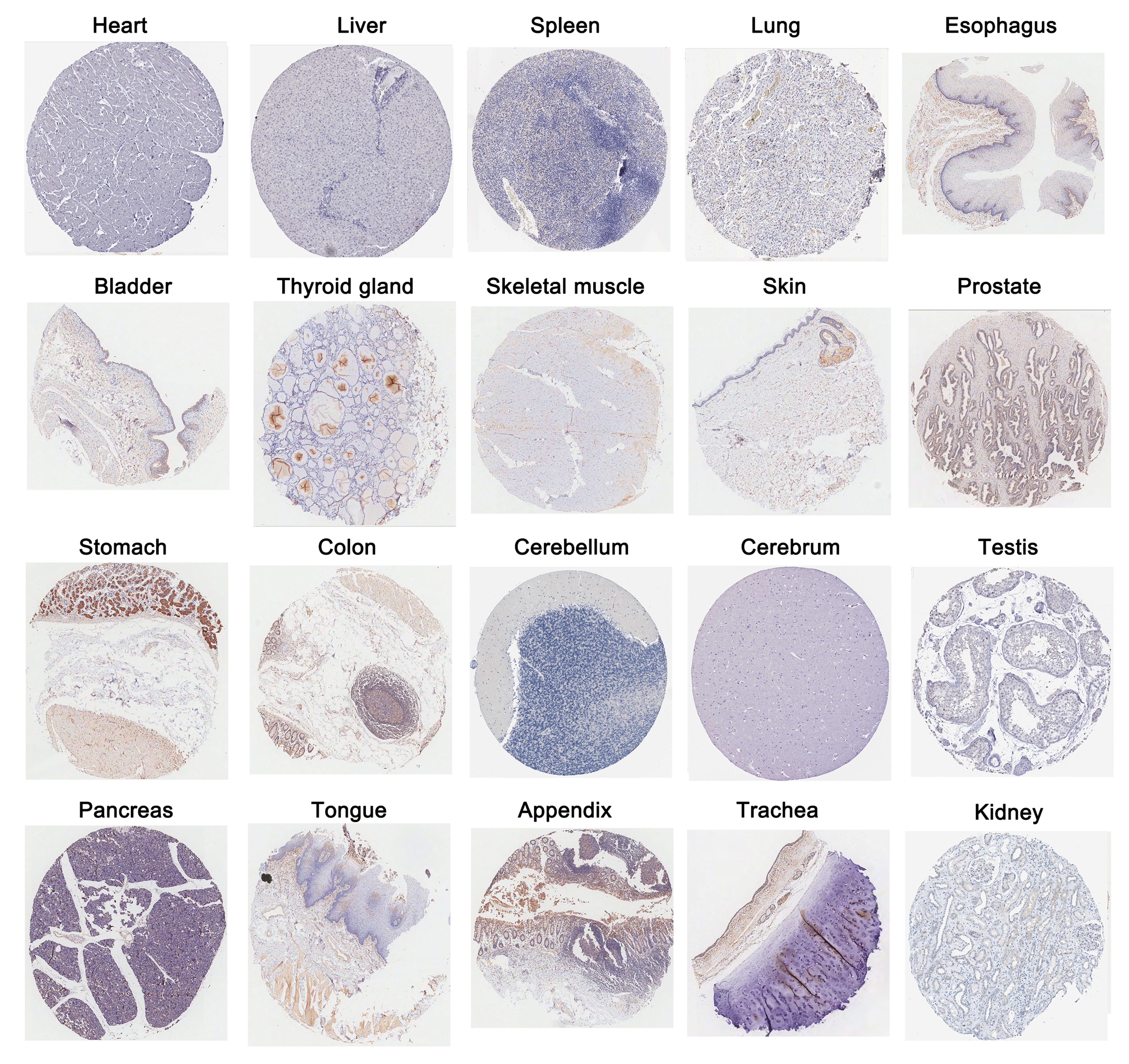

Supplement: Supplementary Figure 7 — Representative micrographs of PTK7 expression in indicated normal human organs assessed by staining with the rabbit polyclonal anti-PTK7 antibody (Invitrogen) at the final concentration of 1 µg/ml. Micrographs are representative of at least 2-3 sections per tissue. Magnification, x 40. [file Image_7.tif]

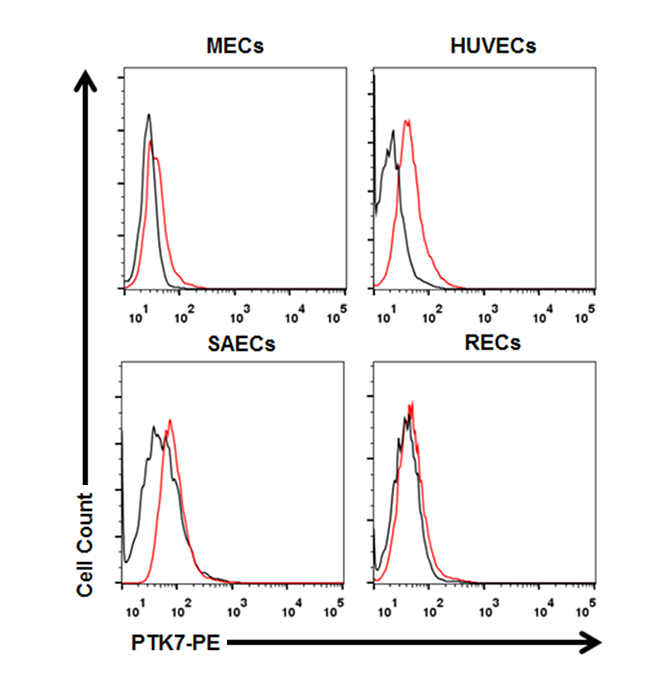

Supplement: Supplementary Figure 8 — PTK7 expression on the primary human normal epithelial cell lines from mammary gland (Mammary Epithelial Cells, MECs), lung (Small Airway Epithelial Cells, SAECs), and kidney (Renal Epithelial Cells, RECs) and human umbilical vein endothelial cells (HUVECs) determined by FACS analysis. Black and red line denote the control (secondary antibody alone) and PTK7 staining respectively. [file Image_8.tif]
