# Supplementary material for: PTK7-Targeting CAR T-Cells for the Treatment of Lung Cancer and Other Malignancies
Source: Front Immunol. 2021 Aug 12;12:665970. doi: 10.3389/fimmu.2021.665970 (PMC8406764; doi:10.3389/fimmu.2021.665970)
Supplement: Supplementary file 9 [file Table_1.docx]

**Table S1. Summary of the PTK7 expression in normal human tissues (Related to Figure S7)**

| **Tissue** | **PTK7 expression** | **Cellular compartment** |
| --- | --- | --- |
| Heart | Negative |  |
| Liver | Negative |  |
| Spleen | Negative |  |
| Lung | Negative | Weak cytoplasmic staining in alveolar septum stroma |
| Esophagus | Negative | Weak cytoplasmic staining of submucosal cells |
| Bladder | Negative | Weak cytoplasmic staining in stroma |
| Thyroid gland | Negative | Suspicious for focal weak cytoplasmic staining of thyroid epithelium |
| Skeletal muscle | Negative | Weak cytoplasmic staining of blood vessel |
| Skin | Negative | Weak cytoplasmic staining of basal keratinocytes |
| Prostate | Weak positive | Weak cytoplasmic staining of epithelium |
| Stomach | Positive | Moderate to strong cytoplasmic staining of gastric epithelium |
| Colon | Weak positive | Weak cytoplasmic staining of epithelium and stroma |
| Cerebellum | Negative |  |
| Cerebrum | Negative |  |
| Testis | Negative |  |
| Pancreas | Negative |  |
| Tongue | Weak positive | Weak cytoplasmic staining in submucosa |
| Appendix | Weak positive | Focal weak to moderate cytoplasmic staining of lymphoid cells |
| Trachea | Weak positive | Weak cytoplasmic staining of epithelial cells |
| Kidney | Weak positive | Weak cytoplasmic staining of tubule epithelial cells |
